# Resistance-Associated NS5A Variants of Hepatitis C Virus Are Susceptible to Interferon-Based Therapy

**DOI:** 10.1371/journal.pone.0138060

**Published:** 2015-09-14

**Authors:** Jun Itakura, Masayuki Kurosaki, Mayu Higuchi, Hitomi Takada, Natsuko Nakakuki, Yoshie Itakura, Nobuharu Tamaki, Yutaka Yasui, Shoko Suzuki, Kaoru Tsuchiya, Hiroyuki Nakanishi, Yuka Takahashi, Shinya Maekawa, Nobuyuki Enomoto, Namiki Izumi

**Affiliations:** 1 Division of Gastroenterology and Hepatology, Musashino Red Cross Hospital, Musashino, Tokyo, Japan; 2 First Department of Internal Medicine, University of Yamanashi, Chuou Yamanashi, Japan; Hokkaido University, JAPAN

## Abstract

**Background & Aims:**

The presence of resistance-associated variants (RAVs) of hepatitis C virus (HCV) attenuates the efficacy of direct acting antivirals (DAAs). The objective of this study was to characterize the susceptibility of RAVs to interferon-based therapy.

**Methods:**

Direct and deep sequencing were performed to detect Y93H RAV in the NS5A region. Twenty nine genotype 1b patients with detectable RAV at baseline were treated by a combination of simeprevir, pegylated interferon and ribavirin. The longitudinal changes in the proportion of Y93H RAV during therapy and at breakthrough or relapse were determined.

**Results:**

By direct sequencing, Y93H RAV became undetectable or decreased in proportion at an early time point during therapy (within 7 days) in 57% of patients with both the Y93H variant and wild type virus at baseline when HCV RNA was still detectable. By deep sequencing, the proportion of Y93H RAV against Y93 wild type was 52.7% (5.8%– 97.4%) at baseline which significantly decreased to 29.7% (0.16%- 98.3%) within 7 days of initiation of treatment (p = 0.023). The proportion of Y93H RAV was reduced in 21 of 29 cases (72.4%) and a marked reduction of more than 10% was observed in 14 cases (48.7%). HCV RNA reduction was significantly greater for Y93H RAV (-3.65±1.3 logIU/mL/day) than the Y93 wild type (-3.35±1.0 logIU/mL/day) (p<0.001).

**Conclusion:**

Y93H RAV is more susceptible to interferon-based therapy than the Y93 wild type.

## Introduction

Hepatitis C virus (HCV) is the leading cause of chronic hepatitis, liver cirrhosis and hepatocellular carcinoma worldwide [1]. Direct acting antivirals (DAAs) that inhibit the HCV proteins necessary for replication have improved the rate of successful HCV eradication [2]. One of the major drawbacks of DAAs-based therapy is the resistance-associated variants (RAVs) which emerge after the failure of DAAs-based therapy [3,4]. These RAVs may attenuate the efficacy of DAAs of the same class, thus the EASL guidelines recommend not to retreat with the same class of DAA in patients who failed prior DAA based therapy [5].

Among three classes of DAAs, NS5A inhibitors are key drugs included in most of the DAAs-based combination therapies [6–9]. Among RAVs for NS5A inhibitors in genotype 1b, Y93H RAV is most frequently detected, with a high level of resistance [10–12]. The Y93H RAV could be induced by the failure of NS5A inhibitor-based therapy but are also present in a substantial portion of patients naïve to DAAs. In our previous report, Y93H RAV was present in 20% of patients naïve to DAAs [13]. The presence of these natural Y93H RAVs at baseline attenuates the efficacy of Daclatasvir (NS5A inhibitor) and Asunaprevir (NS3 protease inhibitor) combination therapy, where the sustained virological response (SVR) rate was 91–97% in patients without RAVs but 39–44% in patients with the Y93H RAV [6,14].

Pegylated interferon (IFN) plus ribavirin (PR) therapy, with or without a combination of NS3 or NS5B inhibitors, may be one of the treatment options in patients with detectable Y93H RAV. However, the efficacy of PR-based therapy with or without DAAs for the Y93H RAV, and the differences in the susceptibility to IFN between Y93H RAV and the Y93 wild type, have not been studied. The aim of this study was to characterize the susceptibility of Y93H RAV to IFN-based therapy by analyzing the changes in the proportion of the Y93H RAV within individuals during therapy.

## Study Design

### Patients

Serum was obtained from a total of 29 genotype 1b patients who had not been exposed to NS5A inhibitor, had detectable Y93H RAV at baseline and were treated by a combination of Simeprevir (SMV) and 24 weeks of PR (SMV/PR) therapy. The clinical backgrounds of the patients are shown in Table 1. In these patients, there were no case with RAV at A156 or R155. One case with D168E RAV at baseline achieved SVR. Two cases had L31M RAV at baseline but both cases achieved SVR by the therapy. All patients fulfilled the following criteria: HBV negative, HIV negative and without other causes of hepatitis, such as primary biliary cirrhosis, autoimmune hepatitis and alcoholic liver disease. Written informed consent was obtained from each patient.

**Table 1:** Baseline characteristics.

Number of cases	29
Age	63.7 ± 9.6
Male / Female	14 / 15
AST (U/L)	52.0 ± 28.7
ALT (U/L)	55.2 ± 31.7
Platelet (x10^-9^/L)	126 ± 56.3
Albumin (g/dl)	3.9 ± 0.37
AFP (ng/ml)	11.9 ± 29.0
Histological diagnosis (METAVIR score)	
Activity score (0-1/ 2/ 3)	14/ 12/ 1
Fibrosis stage (0-1/ 2/ 3–4)	14/ 5/ 10
IL28B (rs8099917) (TT/ TG or GG)	28/ 1
Prior treatment (Naïve / Relapse / NR)	10/ 12/ 7
Treatment outcomes (SVR/ Relapse / NR)	25/ 0/ 4

AST, aspartate aminotransferase’

ALT, alanine aminotransferase’

AFP, alpha-fetoprotein’

PR therapy, pegylated interferon plus ribavirin therapy’

RAV, resistance-associated variants’

SMV/PR therapy; Simeprevir plus pegylated interferon / ribavirin therapy’

TVR, telaprevir,

SVR; sustained virological response

Serum samples were obtained at baseline and at early points after the start of treatment (within 7 days). In 4 patients with breakthrough or relapse, serum was also obtained at the time of the breakthrough or relapse and at least 3 months after the end of treatment. This study protocol conformed to the ethical guidelines of the Declaration of Helsinki and was approved by the institutional ethics review committee of Musashino Red Cross Hospital (approval number 499).

### Analysis by direct sequencing

Direct sequencing was used to detect Y93H RAV in the NS5A region, as described previously [13]. Briefly, viral RNA was extracted from serum, reverse-transcribed and amplified by the two-step nested PCR method. The PCR products were purified and sequenced using an automated DNA sequencer (3730xl DNA Analyzer, Applied Biosystems). Each sequence was confirmed for the sense and anti-sense strands. If the Y93H RAV signal was detected in more than 10% of the total sequence, it was regarded as positive.

### Analysis by deep sequencing

Deep sequencing was performed to quantify the proportion of Y93H RAV against the Y93 wild type within each patient. The methods of extraction of viral RNA and nested PCR were the same as for direct sequencing except for the second-round PCR was performed with a primer including 10 nucleotide barcode tag. After the Emulsion PCR of the nested PCR product, pyrosequencing was carried out using the GS FLX sequencing system (454 Life Sciences, Roche, Branford, CT). Low-quality reads were removed by Quality Filter of GS RunProcessor and a GS Amplicon Variant Analyzer 2.8 was used for the analysis.

### Statistical analysis

Statistical analyses were performed using the SPSS statistics version 18.0 (SPSS Inc, Chicago, IL, USA). A p value of <0.05 was considered statistically significant.

## Results

### Detection of Y93H RAV by direct sequencing at baseline and during SMV/PR therapy

Direct sequencing was performed to detect the Y93H RAV in the NS5A region in 29 patients. At baseline, Y93H RAV and the Y93 wild type were detected as a mixture in 21 of 29 patients: Y93H RAV was detected as a minor variant (wild>H) in 9 cases (case no. 1–9) and as dominant (H>wild) in 12 cases (cases no.10-21). At an early time during SMV/PR therapy (within 7 days of the initiation of therapy) when HCV RNA was still positive, Y93H RAV became undetectable and Y93 wild type prevailed in 6 of 9 cases with ‘wild>H’ (case no.1-4, 8, and 9) and in 3 of 12 cases with ‘H>wild’ (case no.10, 11, and 20), while the proportion of Y93H RAV decreased and the Y93 wild type became dominant in 3 of 12 cases with ‘H>wild’ (case no.12, 13, and 21) (Table 2). Overall, the Y93H RAV became undetectable or its proportion decreased in 57% of patients with both Y93H and the wild type at baseline. Among 8 cases with Y93H RAV alone at baseline (case no.22-29), the Y93 wild type became detectable in one case. The overall rate of SVR was 86.2%.

**Table 2:** Detection of Y93H RAV by direct sequencing at baseline and during SMV/PR therapy.

	(dominant/minor variants)					
Case	at baseline	during treatment*	at relapse/ breakthrough	after treatment**	Number of cases	Outcomes
1–4	wild>H	wild			4	SVR
5, 6	wild>H	wild>H			2	SVR
7	wild>H	H			1	SVR
8	wild>H	wild	wild	H>wild	1	BT
9	wild>H	wild	wild	wild>H	1	BT
10, 11	H>wild	wild			2	SVR
12, 13	H>wild	wild>H			2	SVR
14–18	H>wild	H>wild			5	SVR
19	H>wild	H			1	SVR
20	H>wild	wild	wild	H>wild	1	BT
21	H>wild	wild>H	wild	H>wild	1	Relapse
22	H	H>wild			1	SVR
23–29	H	H			7	SVR

BT, breakthrough;

SVR, sustained virological response

*within 7 days of the initiation of treatment, when HCV RNA was still detectable,

**at least 3 months after the end of treatment

### The proportion of Y93H RAV, quantified by deep sequencing at baseline and during SMV/PR therapy

**Fig 1:**
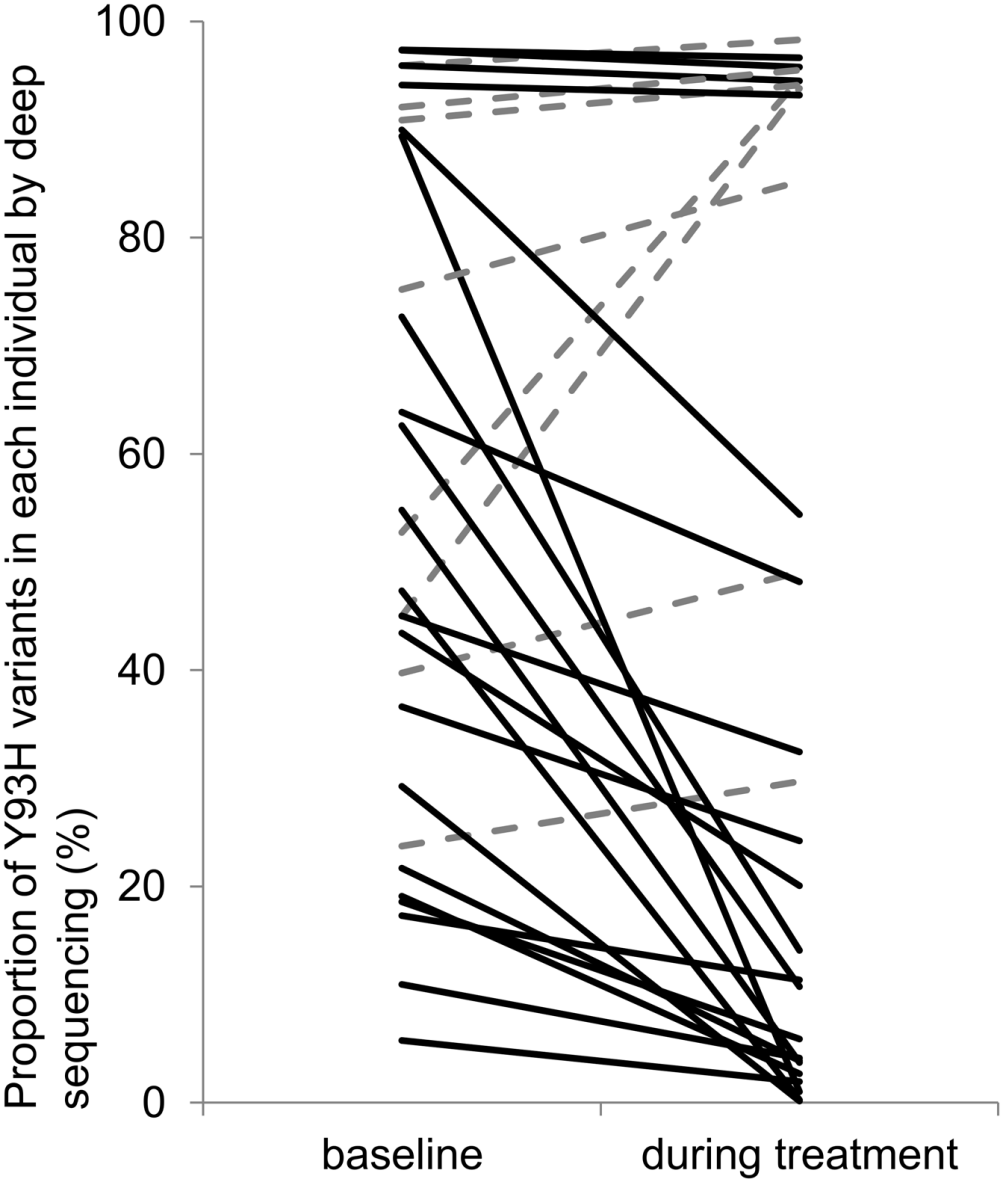
Changes in the proportion of Y93H RAV within each individual. The proportion of Y93H RAV over the Y93 wild type within each patient was determined by deep sequencing at baseline and at an early time point during SMV/PR therapy (within 7 days). The mean proportion of Y93H RAV was 52.7% at baseline and 29.7% during therapy (p = 0.023). The proportion of Y93H was reduced in 21 of 29 cases (72.4%, solid lines). In contrast, Y93H percentages increased in 8 cases (27.6%, broken lines).

Deep sequencing was performed using the samples above to quantify the proportion of Y93H RAV against Y93 wild type within each patient. Fig 1 shows the changes in the proportion of Y93H RAV from baseline to early time point during SMV/PR therapy (within 7 days of the initiation of therapy). Overall, the mean proportion of Y93H RAV was 52.7% (5.8%- 97.4%) at baseline and decreased to 29.7% (0.16%- 98.3%) within 7 days of the initiation of treatment (p = 0.023, paired-t test). For each individual, the proportion of Y93H RAV was reduced in 21 of 29 cases (72.4%) and a marked reduction of more than 10% was observed in 14 cases (48.7%). The proportion of Y93H variants decreased from 89.4% to 1.0% in the most prominent case. Two cases had L31M RAV at baseline but both cases achieved SVR by the therapy. There were no significant changes in the proportion of L31M RAV during therapy in both cases (90% to 92% in one case, and 10% to 8% in another).

### Changes in the proportion of Y93H RAV in 4 cases with breakthrough or relapse

**Fig 2:**
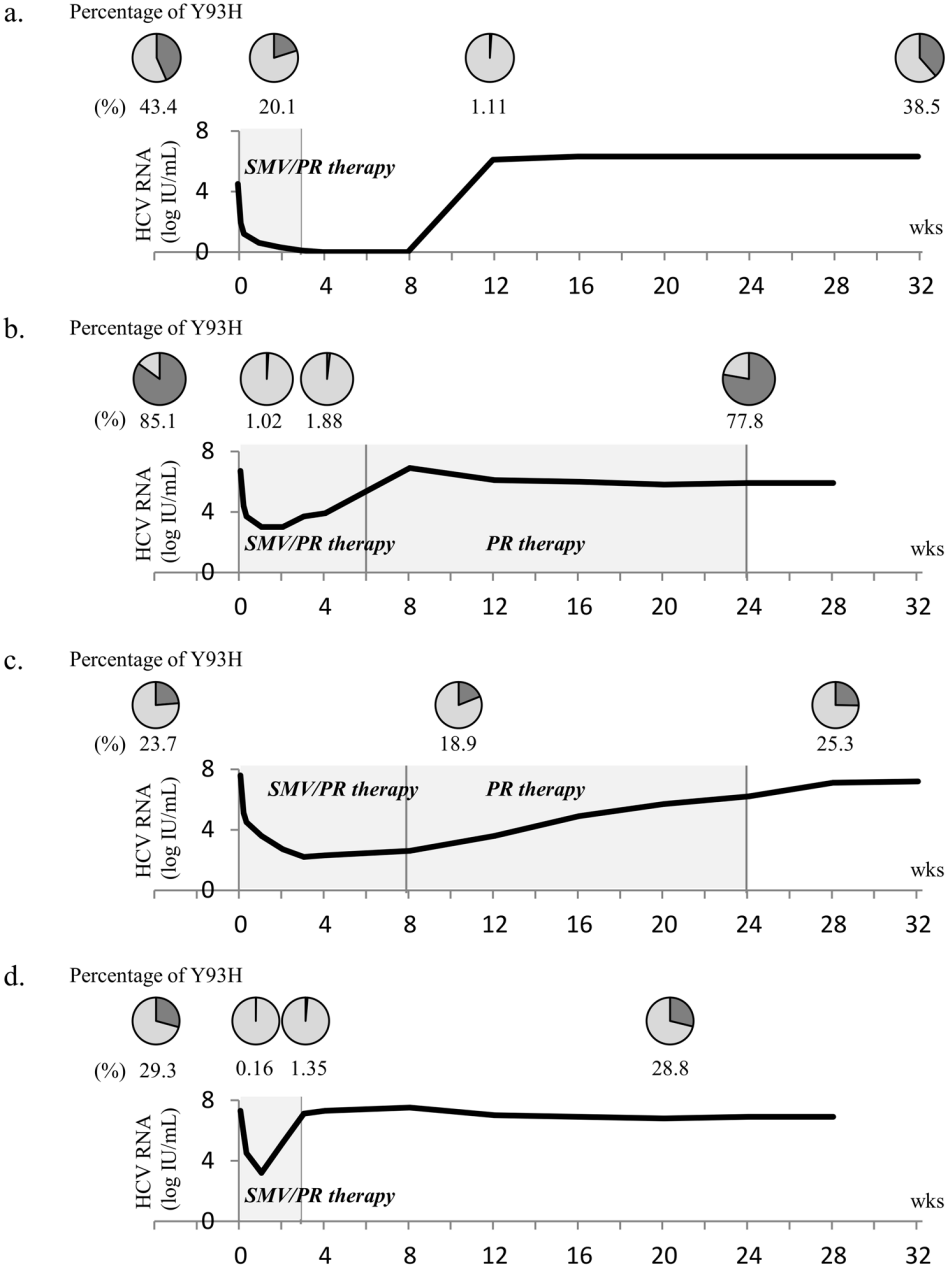
Changes in the proportion of Y93H RAV in 4cases with breakthrough or relapse. Deep sequencing was performed in 4 patients with relapse (a) or breakthrough (b, c, d) to quantify the proportion of Y93H RAV against the Y93 wild type. In two cases, PR therapy was continued up to 24 wks after stopping SMV (b and c). The proportion of Y93H RAV decreased during SMV/PR therapy and at the time of breakthrough/relapse compared to baseline but recovered to the baseline level at follow up. PR therapy; pegylated interferon plus ribavirin therapy, SMV/PR therapy; Simeprevir plus pegylated interferon / ribavirin therapy

SMV/PR therapy was stopped due to breakthrough in 3 cases and 1 patient experienced a relapse after stopping SMV/PR therapy due to an adverse event. Deep sequencing was performed in these 4 patients to quantify the proportion of Y93H RAV against the Y93 wild type at the time of breakthrough or relapse and at 3 months after the end of SMV/PR therapy. Compared to baseline, the proportion of Y93H RAV had decreased at an early time point during therapy and at the time of breakthrough or relapse. However, the proportion of Y93H RAV returned to the baseline level at 3 months after the termination of therapy (Fig 2). In two cases, PR therapy was continued up to 24 weeks after stopping SMV (Fig 2b and 2c). The proportion of Y93H RAV had decreased during SMV/PR therapy and at the time of breakthrough/relapse compared to baseline, but recovered to the baseline level despite continued PR therapy. The D168 RAVs appeared in all cases at treatment failure. There was no case with emerging L31 RAV at treatment failure.

### HCV RNA reduction rate of Y93H RAV versus the Y93 wild type during SMV/PR therapy

**Fig 3:**
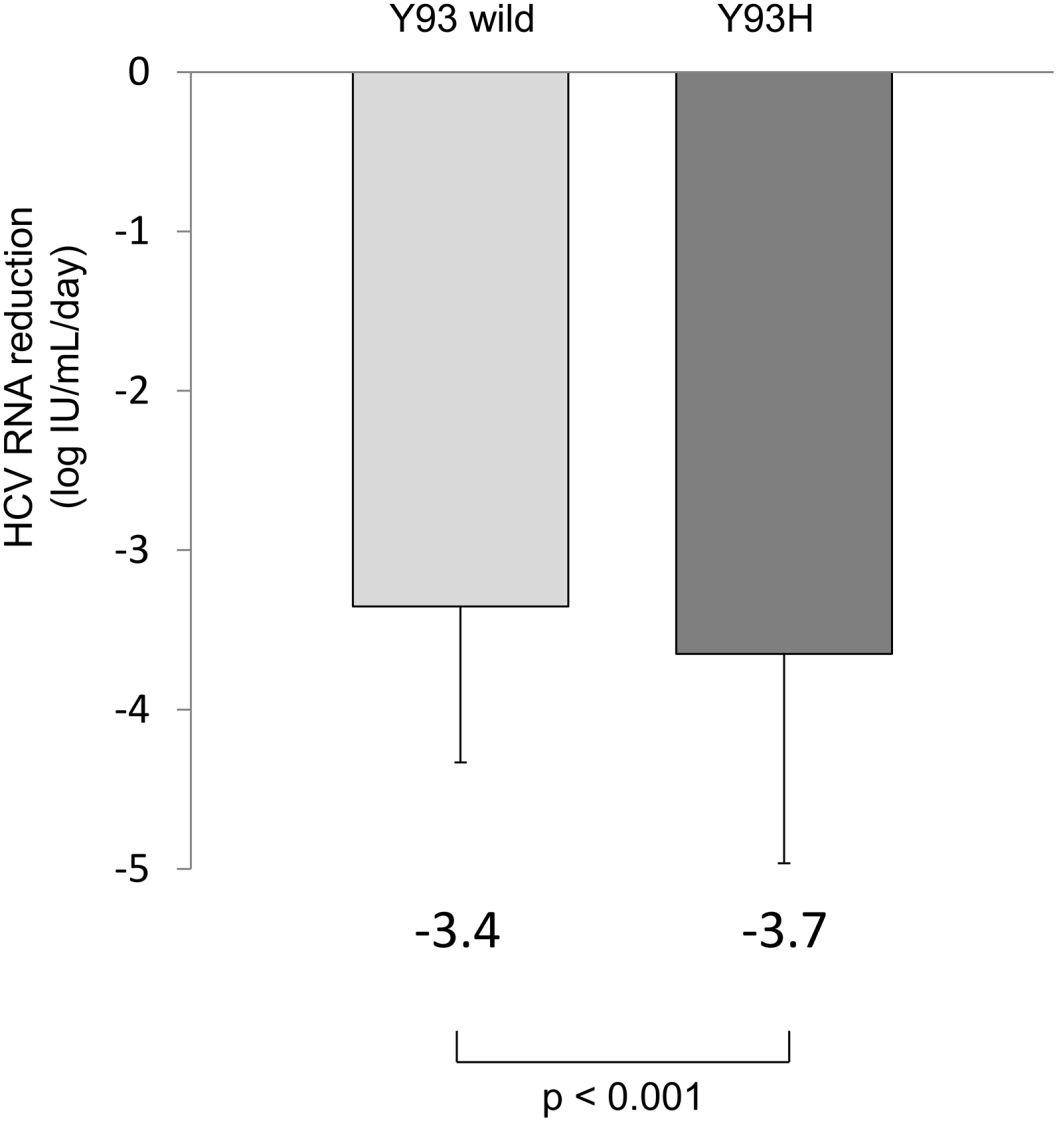
HCV RNA reduction of Y93H RAV versus the Y93 wild type during SMV/PR therapy. Reduction of HCV RNA from baseline to an early time point during SMV/PR therapy was determined for Y93H RAV and the Y93 wild type. Within 7 days of the initiation of therapy, HCV RNA reduction was significantly greater for Y93H RAV (-3.7 ± 1.3 logIU/mL/day) than the Y93 wild type (-3.4 ± 1.0 logIU/mL/day) (p<0.001).

Titers of HCV RNA for Y93H RAV and the Y93 wild type were calculated by multiplying the total HCV RNA by the proportion of each variant. Based on this calculation, reduction of HCV RNA from baseline to the early time point during SMV/PR therapy was determined for Y93H RAV and the Y93 wild type. Within 7 days of the initiation of therapy, HCV RNA reduction was significantly greater for the Y93H RAV (-3.7 ± 1.3 logIU/mL/day) than the Y93 wild type (-3.4 ± 1.0 logIU/mL/day) (p<0.001) (Fig 3).

## Discussion

In this study, we evaluated the dynamic changes in the proportion of the Y93H RAV over the Y93 wild type within 7 days of the start of SMV/PR therapy. The result of the direct sequencing and deep sequencing revealed that the proportion of Y93H RAV against Y93 wild type decreased significantly within individual patients. By calculation, the rate of HCV RNA reduction was greater for Y93H RAV than the Y93 wild type. Taken together, these results indicate that Y93H RAV is more susceptible to interferon-based therapy than the Y93 wild type.

The Y93H RAV of the NS5A region was selected as a target of analysis for the following reasons: First, NS5A inhibitors are included in most DAAs-based combination regimens. Second, the Y93H RAV is detectable in up to 20% of DAA naïve genotype 1b patients [13, 18]. Third, the Y93H RAV is highly resistant to various NS5A inhibitors [6, 17]. Fourth, the Y93H RAV is frequently induced by failure of NS5A inhibitor-based therapy [6, 15–17]. Lastly, NS5A RAVs such as Y93H that emerge after the failure of NS5A inhibitor-based therapy are replication fit and could not cleared spontaneously [15, 16]. The effect of Y93H RAV on treatment outcome and the natural history of emergent Y93H RAV have been most intensively characterized in studies of Daclatasvir and Asunaprevir combination therapy [6,15–17]. Based on these findings, the HCV treatment guidelines of the Japanese Society of Hepatology recommend not to treat patients with Y93H RAV with Daclatasvir and Asunaprevir combination therapy, and the EASL guidelines recommend not to use the same class of DAA in patients who failed prior DAA-based therapy [5].

On this basis, PR therapy with or without a combination of DAAs may be one of the treatment options for patients with detectable Y93H RAV. Currently available options include PR plus SMV or Vaniprevir or Telaprevir in Japan, while PR plus Sofosbuvir may be another option in the USA and European countries. In our study, the overall rate of SVR in patients with Y93H RAV at baseline was as high as 86.2% following SMV/PR therapy, which is in contrast to the reported lower SVR rate of 39–44% following treatment with a combination of Daclatasvir and Asunaprevir. Together with the finding that Y93H RAV is more susceptible to interferon-based therapy than the Y93 wild type, the results of our study provided a proof of concept that the presence of an RAV to an NS5A inhibitor does not attenuate the efficacy of an NS3 inhibitor or PR, and that combination of SMV plus PR could be an alternative treatment option against Y93H RAV.

The underlying mechanism of the IFN susceptibility of Y93H RAV is unclear. Previous studies revealed that Y93H RAV was less frequently detected in patients with the unfavorable IL28B genotype non-TT (rs8099917) [13,18]. In other words, Y93H RAV may be more fit to replicate in the liver of IL28B TT patients than those with non-TT. The most prominent difference in the liver environment between the IL28B TT and non-TT is the hepatic expression of IFN stimulated genes. The hepatic expression level of IFN stimulated genes is higher in non-TT, probably reflecting the basal activation of the intrinsic IFN system [19]. One possible hypothesis is that the Y93 wild type has the ability to prevail over Y93H RAV under a high level of intrinsic IFN in IL28B non-TT. The concept of this hypothesis goes hand-in-hand with the result of the present study that Y93H RAV is more susceptible to externally administered IFN.

The viral load reduction rate during the first few days of IFN-based therapy has been reported to reflect the theoretical replication rate of virus [20,21]. Since HCV RNA reduction was significantly greater for Y93H RAV than the Y93 wild type during the first 7 days of treatment, it may be possible that the efficiency of virus replication under the influence of IFN is lower for Y93H RAV than the Y93 wild type. The analysis of 4 patients with breakthrough or relapse revealed that Y93H RAV was suppressed by SMV/PR therapy but returned to baseline after discontinuation of therapy. Thus, the replication efficiency of Y93H is apparently influenced by the presence of SMV/PR. NS5A is a non-structural protein necessary for HCV replication [22] and the known pleiotropic functions of NS5A include binding to HCV RNA [23–25], formation of HCV non-structural protein aggregates, formation of double membrane vesicles for HCV RNA replication [26,27], involvement in packaging and assembly of the virus [28,29] and influence on innate and acquired immunity [30–33]. Thus, mutation in NS5A has the potential to affect virus replication efficiency. Position Y93 is located in domain 1 of NS5A, where the NS5A protein forms a dimer [24,34,35], NS5A inhibitors bind [25], and is close to the site of anchorage to the ER membrane [36]. How the Y93H RAV may affect the lifecycle of HCV remains to be elucidated. Future *in vitro* study comparing the replication capacity of Y93H RAV and the Y93 wild type is necessary.

In this study, the proportion of Y93H against Y93 wild type decreased significantly during treatment when analyzed as a whole, and actually, proportion of Y93H decreased in 21 out of 29 patients. However, proportion of Y93H RAV increased in 8 patients. Of these eight cases, more than 10% increment was observed in only three cases (10%). There were no significant differences in the various factors including clinical characteristics, laboratory data, viral load, or treatment response between patients with and without Y93H decrease. Therefore, the mechanisms of Y93H increase in these patients could not be defined. It may be possible that hepatitis C viral sequence other than Y93 position may have affected the sensitivity to interferon in these cases leading to selection of clones. Further study is needed to elucidate the precise mechanism.

In conclusion, Y93H RAV is more susceptible to interferon-based therapy than the Y93 wild type. This is a proof of concept that the presence of RAV to NS5A inhibitors does not attenuate the efficacy of NS3 inhibitor or PR, and that the combination of SMV and PR could be an alternative treatment option against Y93H RAV, other than combination therapy with DAAs.
